# Effects of concentrated long-chain omega-3 polyunsaturated fatty acid supplementation before radical prostatectomy on prostate cancer proliferation, inflammation, and quality of life: study protocol for a phase IIb, randomized, double-blind, placebo-controlled trial

**DOI:** 10.1186/s12885-017-3979-9

**Published:** 2018-01-10

**Authors:** Marie-Hélène Guertin, Karine Robitaille, Jean-François Pelletier, Thierry Duchesne, Pierre Julien, Josée Savard, Isabelle Bairati, Vincent Fradet

**Affiliations:** 1 Oncology Unit, Centre de recherche du CHU de Québec – Université Laval - L’Hôtel-Dieu de Québec, 6 rue McMahon, Québec, QC Canada; 2 Mathematics and Statistics Department, Université Laval, 1045 avenue de la médecine, Bureau, Québec, QC 1056 Canada; 3 Endocrinology and Nephrology Unit, Centre de recherche du CHU de Québec – Université Laval - CHUL, 2705, boulevard Laurier, Québec, QC Canada

**Keywords:** Prostate cancer, Omega-3, Proliferation, Inflammation, Quality of life

## Abstract

**Background:**

Prostate cancer is the most commonly diagnosed cancer in north-American men. Few dietary or lifestyle interventions have been tested to prevent prostate cancer progression. Omega-3 fatty acid supplementation represents a promising intervention for prostate cancer patients. The aim of the study is to evaluate the effects of long-chain omega-3 polyunsaturated fatty acids (LCn3), more precisely eicosapentaenoic acid monoacylglyceride (MAG-EPA) supplementation, on prostate cancer proliferation, inflammation mediators and quality of life among men who will undergo radical prostatectomy.

**Methods/design:**

We propose a phase IIb, randomized, double-blind placebo-controlled trial of MAG-EPA supplementation for 130 men who will undergo radical prostatectomy as treatment for a prostate cancer of Gleason score ≥ 7 in an academic cancer center in Quebec City. Participants will be randomized to 6 capsules of 625 mg of fish oil (MAG-EPA) per capsule containing 500 mg of EPA daily or to identically looking capsules of high oleic acid sunflower oil (HOSO) as placebo. The intervention begins 4 to 10 weeks prior to radical prostatectomy (baseline) and continues for one year after surgery. The primary endpoint is the proliferative index (Ki-67) measured in prostate cancer cells at radical prostatectomy. A secondary endpoint includes prostate tissue levels of inflammatory mediators (cytokines and proteins) at time of radical prostatectomy. Changes in blood levels of inflammatory mediators, relative to baseline levels, at time of radical prostatectomy and 12 months after radical prostatectomy will also be evaluated. Secondary endpoints also include important aspects of psychosocial functioning and quality of life such as depression, anxiety, sleep disturbances, fatigue, cognitive complaints and prostate cancer-specific quality of life domains. The changes in these outcomes, relative to baseline levels, will be evaluated at 3, 6, 9 and 12 months after radical prostatectomy.

**Discussion:**

The results from this trial will provide crucial information to clarify the role of omega-3 supplementation on prostate cancer proliferation, inflammation and quality of life.

**Trial registration:**

ClinicalTrials.gov Identifier: NCT02333435. Registered on December 17, 2014. Last updated September 6, 2016.

**Electronic supplementary material:**

The online version of this article (10.1186/s12885-017-3979-9) contains supplementary material, which is available to authorized users.

## Background

Prostate cancer (PCa) is a significant health problem worldwide. In Canada, 1 out of 8 men is expected to develop PCa in their lifetime and 1 in 27 will die from it [1]. Men diagnosed with intermediate risk PCa usually undergo radical prostatectomy (RP) or radiation therapy and have uncertain prognoses [1] and many side effects [2, 3]. For these patients, dietary and lifestyle interventions are considered promising strategies to improve health and quality of life [4].

### Omega-3 fatty acids

Long-chain omega-3 polyunsaturated fatty acids (LCn3), eicosapentaenoic acid (EPA) and docosahexanenoic acid (DHA), mainly found in seafood and fatty fish, might help lower PCa incidence and/or delay its progression [4–8]. However, some reviews reported no association [9–12] or mixed associations depending on LCn3 subtypes [13, 14]. It is important to note that observational studies are often limited by multiple sources of bias and the difficulty of estimating LCn3 intakes. LCn3 levels measured in red blood cells reflect the diet over the last 3 months [15]. However, studies that evaluated dietary LCn3 from biomarkers, assessed LCn3 in the plasma [9–11, 16–18], a measure that reflects the diet of the past few days [15]. Interestingly, we have recently measured LCn3 in the targeted prostate tissue during active surveillance of patients with a low-grade PCa. We observed a significant protective association between higher levels of EPA and a lower risk of progression to high-grade PCa [19].

### Proliferation

Nuclear Ki-67 is a protein expressed in all proliferative phases of the cell cycle [20–23].The proliferation rate of normal prostatic epithelial cells being very low, Ki-67 is mainly expressed in PCa cells and this proliferative index is considered an important prognostic factor for PCa patients [24–27]. A Phase II randomized controlled trial (RCT) comparing a low fat diet enriched with fish oil to a western diet, in 55 men, showed that prostatic Ki-67 expression was significantly reduced in the low-fat diet/fish oil group [28]. Flaxseed supplementation before RP was also associated with downregulation of Ki-67 in another RCT [29]. However, in these studies, proliferation was not the primary endpoint.

### Inflammation

Inflammation is a highly ordered, controlled and short-lived response to infection or injury. Tumors are often viewed as “wounds that do not heal” and can prevent the proper regulation of the resolution phase of inflammation, thus taking advantage of the inflammatory process for their own benefits. The microenvironment surrounding tumors can produce and secrete several cytokines and growth factors that promote proliferation and minimize apoptosis, thus driving carcinogenesis [30, 31]. Over-expression of several inflammatory mediators in prostate tissue (e.g. IL-1 [32, 33], IL-6 [32–34], TGF-β [35], TNF-α [32]) and blood (e.g. IL-6 [36], IL-7 [37] and IL-15 [37]) has been observed in PCa patients or PCa patients with progression.

LCn3, particularly EPA, have beneficial effects on systemic inflammation via modulation of the immune system, increase phagocytic activity, disruption of TLR signaling cascade and production of anti-inflammatory eicosanoids [38]. These effects are mediated by their incorporation into the plasmatic membrane. Previous studies have assessed the changes of only a limited range of systemic inflammatory mediators after omega-3 interventions, including IL-6 [39–43], IL-1β [40, 41, 43–46] and TNF-α [42–44]. However, the effects of nutritional interventions on prostate tissue inflammation has yet to be examined using a RCT design.

### Quality of life and psychosocial functioning

PCa and its treatment are associated with significant psychological distress. Large-scale epidemiological studies on psychological disorders in the context of PCa are sparse. Nonetheless, in a study conducted by our team in 861 patients treated for PCa, we found that 17.0% exhibited clinical levels of depression, while 23.7% of the patients had clinical levels of anxiety [47]. Moreover, we observed sexual difficulties, sleep impairments and fatigue in 70.5%, 31.9%, and 18.5% of the patients, respectively.

Epidemiological studies have shown associations between a greater annual fish intake and lower depression rates [48–50]. A RCT conducted in medical students (with no psychiatric disorder), comparing a 12-weeks LCn3 supplementation to a placebo, showed a 20% reduction of anxiety symptoms [51]. A recent study found no significant effect of omega-3 supplementation on sleep quality [52], while a study of 633 breast cancer survivors showed that a higher intake of omega-6 relative to omega-3 was associated with a higher risk of fatigue [53]. These questions remain to be investigated in PCa patients using a RCT.

### Rationale

Epidemiological studies point to a possible role of environmental factors, especially diet, in PCa incidence and progression. Evidence also suggests that an LCn3-rich diet may be beneficial to cancer patients through the modulation of cancer cell proliferation, inflammation, psychosocial functioning and quality of life. However, well conducted RCT assessing the effects of LCn3 on all these outcomes critical to PCa, are lacking. Therefore, a randomized, double-blind, placebo-controlled trial was initiated to examine the specific roles of LCn3 sub-type EPA on the biology and treatment consequences of PCa.

### Study objectives

We hypothesize that supplementation with EPA monoacylglyceride (MAG-EPA), beginning 6 weeks (range 4 to 10 weeks) before RP and for a year after RP, will have measurable effects on selected PCa outcomes. The specific objectives are as follows:

### Primary objective

To determine the effect of daily MAG-EPA supplementation compared to placebo, on the proliferative index (nuclear Ki-67 expression) of prostate cancer cells from the RP specimen.

### Secondary objectives

To determine the effect of the intervention, compared to placebo, on the targeted tissue expression of inflammatory mediators measured in the prostate tissue from the RP specimen.

To determine the effects of the intervention, compared to placebo, on blood levels, relative to baseline levels (before beginning of the intervention), of inflammatory mediators (cytokines and proteins), at the time of RP and one year after RP.

To assess the effect of the intervention, compared to placebo, on psychosocial functioning and quality of life outcomes, relative to baseline levels, at the time of RP and during the year following RP.

## Methods/design

### Trial design and setting

**Fig. 1:**
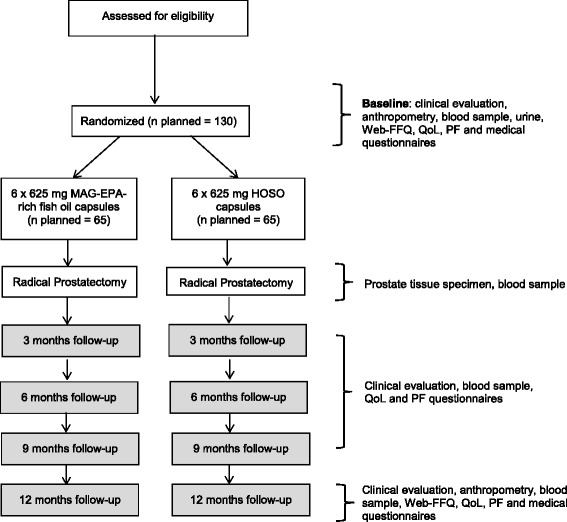
Randomized Controlled Trial flow chart. 130 men diagnosed with intermediate-risk PCa treated by RP are being randomized either to LCn3 supplementation (MAG-EPA) or placebo. Intervention starts 6 weeks (acceptable range 4–10 weeks) before radical prostatectomy (RP) and ends 12 months after RP. PCa: Prostate cancer; Web-FFQ: online Food frequency questionnaire; QoL: Quality of life; PF: Psychosocial functioning; LCn3: Long chain omega-3 fatty acids; RP: Radical prostatectomy; MAG-EPA: Monoglycerides of eicosapentaenoic acid; HOSO: High oleic acid sunflower oil

A phase IIb, randomized, double-blind, placebo-controlled trial will be conducted at the Centre Hospitalier Universitaire (CHU) de Québec – Université Laval, a supra-regional center with high surgical volume for urological cancer, particularly for prostate cancer (>350 RP per year). The parallel study design is presented in Fig. 1.

### Recruitment

The study is advertised in the clinic with posters and the urologists will be reminded regularly of the study. At time of diagnosis, the urologist will discuss the different treatment options with the patient. When RP is chosen as part of the treatment, the patient will be informed of the study. Then, the research nurse will present the study information and the consent form. The patient will have the needed time to decide whether he wants to participate or not to the study.

### Patient population and eligibility criteria

#### Inclusion criteria

Patients must be 18 or older, give informed consent and have chosen RP for treatment of a PCa with a Gleason score ≥ 7.

#### Exclusion criteria

Patients are not eligible if they are intolerant or allergic to fish or sunflower seeds or if they have a diagnosis of bipolar disorder.

#### Washout period

Patients already taking omega-3 supplements can participate after a washout period of at least 8 weeks before randomization. Other dietary supplements must be stopped before randomization.

### Randomization/concealment/blinding

Patients will be randomized to the intervention or placebo group 6 weeks (acceptable range 4 to 10 weeks) prior to RP. Randomization will take place at the preoperative appointment or an appointment taken specifically for the study. The randomization process will consist of a computer-generated random listing of the treatment arm using a 1:1 allocation. The randomization will be generated by the Clinical research oncology pharmacy, using permuted random blocks of 2 to 8. Patient allocation information will be kept in a binder in a locked room of the pharmacy for the entire study period. Patients, as well as all study personnel, including outcome assessors, and medical doctors will be blinded to treatment allocation and block sizes.

### Intervention

Participants assigned to the intervention will receive, for each intervention day, 6 capsules of 625 mg of fish oil (MAG-EPA) per capsule. The supplementation is highly concentrated in EPA giving a total dosage of 3 g of EPA daily. The novel fish oil formulation is based on monoglycerides containing 89% of LCn3, with 80% EPA. It presents a unique ratio of EPA/DHA of more than 10. Most of the available products have a ratio of less than 2. It also contains approximately 3% omega-6, 3% monounsaturated and 3% saturated fatty acids and have a less pronounced “fishy” taste compared to usual preparations.

Participants assigned to the placebo group will receive, for each intervention day, 6 capsules of identical appearance containing high oleic acid sunflower oil (HOSO). These capsules contain 82% of omega-9 and are poor in omega-3 or omega-6. This is a biologically neutral oil and has thus been used as a placebo in at least 3 LCn3 RCTs [43, 54, 55].

Capsules, for intervention and placebo, are prepared by SCF Pharma, Ste-Luce (Qc), and will be odorless and of identical appearance for both groups. Health Canada approved the RCT protocol and the products used for the intervention and placebo arms.

For both groups, the intervention will start 6 weeks (range 4 to 10 weeks) prior to RP and will be pursued for one year after RP. At randomization, patients receive, by the pharmacy personnel, the amount needed until their first follow-up visit, three months after surgery. They then receive, at every follow-up visit, the amount needed for the next three months until the end of follow-up.

The follow-up and care received by the patients will be the same for both groups.

### Concomitant medication

Health Canada does not have any contraindication for the proposed daily dose [56]. Thus, there will be no contraindication concerning other medications taken by the patients and they will be asked to follow their usual regimen. Medications’ usage will be carefully documented at the initial and subsequent visits.

### Data collection and follow-up

The assessment schedule of the study is presented in Table 1. Patients will be assessed at randomization, surgery and every 3 months until one year after RP.

**Table 1:** Data collection schedule

	Initial visit	Randomization	RP	Post-RP follow-up			
	Eligibility	Intervention (baseline - V0)	V1	V2 3 mo	V3 6 mo	V4 9 mo	V5 12 mo
Consent		X					
Medical history and health behaviors		X					
Concomitant medication/symptom evaluation		X		X	X	X	X
Consultation with uro-oncologist	X			X*	X*	X*	X*
Blood sample including total PSA and lipid profile		X	X	X	X	X	X
Digital rectal exam and urine sample		X					
Radical prostatectomy (RP)			X				
Food frequency questionnaire - FFQ online		X					X
Prostate specific quality of life (IPSS and EPIC-26)		X		X	X	X	X
Hospital Anxiety and Depression Scale (HADS)		X		X	X	X	X
Quality of life (sleep quality, fatigue, cognitive functioning) (ISI, ISF, FACT-COG V3)		X		X	X	X	X
General health and wellbeing (SF-36)		X		X	X	X	X
Fear of recurrence (FCRI)		X		X	X	X	X
Self-reported sleep diary		X	X	X	X	X	X
Therapeutic expectation questionnaire				X			X
Anthropometric measurements^¶^		X					X
Vital signs^£^		X		X	X	X	X

RP: Radical Prostatectomy; V: visit; mo: months; PSA: Prostate Specific Antigen; IPSS: International Prostate Symptoms Score; EPIC: Expanded Prostate Index Composite; HADS: Hospital Anxiety and Depression Scale; ISI: Insomnia Severity Index; FSI: Fatigue Symptoms Inventory; Fatigue Symptoms Inventory and Functional Assessment of Cancer Therapy – Cognitive Function (FACT-COGv3); SF-36: Short-Form Health Survey; FCRI: Fear of Recurrence Inventory

*The consultation with the urologist might be on a different day but at the same period

^¶^Height, weight, hip and waist circumference, body fat measured by skinfold caliper

^£^Temperature, blood pressure and pulse

### Prostate tissue harvesting and biological specimens

At the time of RP, immediately after surgical removal, the prostate will be kept cold (4 °C) and transferred to the pathology unit for evaluation. A complete cross-section of the prostate will be harvested by the pathologist. The cross-section will be divided in four specific quadrants before Optimal cutting temperature (OCT) compound freezing procedure. A small portion of each quadrant (approximately 10 mg) will be harvested from the cross-section and snap frozen separately. All prostate tissues are stored at −80 °C.

50 ml of blood will be collected at indicated time-points (Table 1). Plasma, buffy coat, red blood cells, and serum will be processed from 35 mL of blood collected. All samples will be stored at −80 °C. Urine post-digital rectal examination will be collected at initial visit and stored in several buffers at −80 °C.

Procedures used in this research followed the Standard operating procedures (SOPs) of the Canadian Tissue Repository Network (CTRNet). All biological specimens will be stored at the Centre de recherche du CHU de Québec – Université Laval – L’Hôtel-Dieu de Québec hospital and all patients will have provided consent for long-term storage of their tissue.

Detailed information about samples, volume collected and aliquot is provided in the (see Additional file 1: Table S1).

### Outcomes

#### Primary outcome

The primary outcome is cancer proliferation. After immunostaining for Ki-67, slides will be scanned using an automated slide scanner and the proportion of cancer cells with positive nuclear staining will be automatically quantified using algorithms we previously developed with the Calopix (Tribvn) software [27]. Initial results in PCa tissue from RP specimens at our institution showed a linear correlation between automated quantification and visual evaluation [27]. This PCa proliferative index was also an independent predictor of prostate cancer specific mortality, making this outcome clinically relevant.

#### Secondary outcomes

Many inflammation mediators (Il-2, IL-8, IL-10, IL-12, IFN-γ, to name a few) will be measured in the prostatic tissue and in circulation using a Bio-plex Precision Pro kit (Bio-Rad, Toronto, On). The limit of sensitivity (LoS) ranges from 0.2–2.7 pg/mL, allowing for greater probability of detecting the cytokines of interest, if expressed, than usual multiplexing kits (LoS of 2–5 pg/mL).

Prostate specific aspects of quality of life is assessed using the International Prostate Symptoms Score (IPSS) and the Expanded Prostate Index Composite score (EPIC-26) for which the French-Canadian version has been validated [57, 58]. The IPSS contains 7 questions concerning lower urinary tract symptoms for which severity is scored on a 0–5 points scale. It also contains one quality of life item scored on a scale ranging from 0 to 6. EPIC-26 contains 5 quality of life PCa-specific domains: urinary incontinence, urinary irritation/obstruction, bowel, vitality/hormonal, and sexual function. Each domain is scored on a scale from 0 to 100.

Anxiety and depression are assessed using the French-Canadian version of the Hospital Anxiety and Depression Scale (HADS) for which we observed excellent internal consistency and test-retest reliability [59]. The questionnaire includes 14 items scored on a scale range from 0 to 3, with 7 items assessing anxiety and 7 items assessing depression.

Fatigue related quality of life is assessed using French versions of the Insomnia Severity Index (ISI) [60] and the Fatigue Symptoms Inventory (FSI) [61]. The ISI enquires about sleep quality over the past two weeks and contains 7 items scored on a 0 to 4 scale. Good psychometric properties have been reported [62]. The FSI questionnaire assesses, for the past week, fatigue intensity (4 items), fatigue duration (2 items) and how fatigue interfered with quality of life (7 items). The questionnaire exhibited high internal consistency and construct validity [61].

Cognitive function was also evaluated with the Functional Assessment of Cancer Therapy – Cognitive Function (FACT-COG v3) [63]. The questionnaire contains 37 items covering 4 subscales. The subscale includes Perceived cognitive impairments, Comments from others, Perceived cognitive abilities and Impact on quality of life. Each item is scored on a 5-points Likert scale ranging with a range of 0 to 4. This tool also showed high consistency and validity [63].

To assess cancer-specific anxiety, fear of recurrence is evaluated using the 9 items of the severity subscale of the Fear of Cancer Recurrence Inventory (FCRI) which was validated in french [64]. The items are scored from 0 to 4 and assess the presence, frequency and intensity of thoughts associated with cancer recurrence.

Finally, general wellbeing is evaluated using the French version of the Short-Form Health Survey, SF-36, (SF-36v2 Standard, Canada (French) Version 2.0) [65].

### Confounding variables

Potential confounders will be measured at baseline, before the beginning of the intervention. Age, anthropometry, medical history, cancer stage, clinical and pathological grade (Gleason), alcohol and tobacco use will be assessed.

Physical activity for a typical 7-day period will also be assessed at baseline by the Godin Leisure-Time Exercise Questionnaire [66]. The questionnaire contains 3 items assessing the number of times engaging in mild, moderate and strenuous exercises for at least 15 min. One item evaluates the number of times one engages in any activity long enough to work up a sweat.

Dietary intake in the past month will be measured using a web-based self-administered food frequency questionnaire (web-FFQ), which contains 136 questions and 40 sub-questions covering 8 food categories including the four groups of the Canadian Food Guide. The questionnaire has been specifically developed for the population of Quebec and validated in healthy men [67]. We also specifically validated the assessment of omega-3 intake in a population of PCa patients [68]. Dietary intake will be assessed at baseline and 12 months post-RP.

### Adherence and therapeutic expectations

The pharmacy personnel will monitor adherence by counting remaining pills. The success of blinding will be evaluated using a therapeutic expectations questionnaire administered at 3 and 12 months post-RP.

### Sample size calculation

Sample size analysis was done using a two-sample t-test for a log-normal geometric mean ratio with a two-sided significance level of 0.05, assuming equal variances. Based on published data [28, 29], we assume a coefficient of variation of 0.4. In the study conducted by Aronson et al. [28], a statistically significant reduction of 32% in the proportion of cells expressing Ki-67 was observed in a group receiving a low-fat diet supplemented with fish oil compared to a control group assigned to a Western diet. We determined that, for the primary outcome, a total of 126 patients (63/group) will provide 90% power [69, 70] to detect a mean ratio of the proportions of cancer cells expressing Ki-67 of ≤0.8, i.e. a 20% difference across groups. We estimate ≤3% drop out before RP (occurring in ≤1% at our institution). Based on these estimation, the sample size needed for the trial will be 130 patients (65/group).

Twelve months after RP, we expect a 5 to 10% loss to follow-up, which will affect only secondary outcomes. In absence of published data on the effects of omega-3 supplementation on inflammation mediators, we used Cohen’s *d* to calculate effect sizes [71]. A total sample size of 116 will provide 80% statistical power to detect a moderate or large effect size (≥0.5). This sample size will also be sufficient to detect expected between-groups differences on quality of life dimensions. Indeed, LCn3 supplementation reduced anxiety by 20% (d = 0.51) in one study [51] and it yielded a moderate effect size (d = 0.61) for treating depressive disorders (vs. placebo) in another [72]. For these effect sizes in our final sample, we evaluate a power of 80% to detect an effect for anxiety and a power of 92% to detect an effect for depressive symptoms.

### Statistical analyses

#### Primary outcome

The average proportion of cells expressing Ki-67 in each group will be compared, using the t-test, after transformation of the data if the population is not normally distributed. The Wilcoxon rank sum test will be used if observations are not normally distributed after transformation procedures. Multivariable linear regression will be carried out in the event of unbalanced distributions of important baseline characteristics such as cancer grade and stage, age, baseline inflammatory levels, diet or health behaviors. The intervention leading to the primary outcome is of a relatively short duration (4–10 weeks) and since almost all recruited patients are expected to undergo prostatectomy, few missing data are expected. The analyses will include all randomized patients for whom a ki-67 is measurable.

A *per protocol* analysis will also be carried out as exploratory analyses. These analyses will include participants with a measurable ki-67 and who will have taken at least 80% of intervention or placebo doses.

For all analyses, bilateral *p*-values of less than 0.05 will be considered statistically significant.

#### Secondary outcomes

For the secondary outcome pertaining to prostatic levels of inflammation mediators, the t-tests (or Wilcoxon rank-sum test) will be used to compare levels across groups.

Blood levels of inflammation biomarkers as well as outcomes concerning quality of life are measured at multiple time points. For these analyses, linear mixed models, to take into account for multiple measurements, will be performed.

Confounding factors will also be taken into account in the analyses in the case of unbalanced distribution between groups.

### Subgroup analyses

It is possible that some patients will already have high dietary intake of omega-3. Subgroups analyses will be carried out to explore how baseline levels of omega-3 in the diet can modify the effect of the intervention. The effect of the intervention will be tested for low or high levels of total omega-3 (and LCn3) using intake with an interaction term in the multivariable linear regression. The levels measured in red blood cells, a good proxy to the diet, will also be evaluated. The cutoff will be the recommended dietary intake of 1.6 g daily [73] for total omega-3 and 250 mg for EPA (the equivalent of approximately 8 oz per week of a variety of seafood [74]). For red blood cells levels, the value corresponding to the recommended dietary intake (modeled in this cohort) will be used as a cutoff.

### Ethical considerations

The study protocol has been approved by the ethics committee of CHU de Québec - Université Laval (2012–1012). Written informed consent is obtained from all randomized patients for the study as well as for biobanking of their biological specimens (blood, urine, tissue).

### Adverse events safety monitoring

Many studies have already shown LCn3 safety. Therefore, no major harmful effects are expected. However, adverse events will be monitored at each visit post-prostatectomy using the NIH criteria for adverse events (v4.03, June 2010). An independent safety monitoring board includes a statistician Dr. Benoit Masse (PhD), Head of Applied Clinical Research Unit of CHU Sainte-Justine Research Center, Montréal, Quebec and a prostate cancer and clinical research trial expert, Dr. Fred Saad (MD), Centre Hospitalier de l’Université de Montréal Research Center, Montreal, Quebec.

### Data collection, managing and monitoring

All data is collected at the research center by research professionals. Questionnaires are scanned and validated by a research professional. The data is stored on the network of the Centre de recherche du CHU de Québec – Université Laval following their Standard Operative Procedures to ensure confidentiality. Only the PI and the research professionals implied in the current study will have access to the data and protected by a password. Questionnaires will be stored in a locked filing cabinet which is in a locked room. Data collection follow-up monitoring will be realized on a regular basis by the research team.

## Discussion

This trial is the first to examine the effects of preoperative LCn3 supplementation with Ki-67 as a primary outcome. Moreover, it is, to our knowledge, the first to examine the effects of LCn3 on inflammation and quality of life among PCa patients. Well conducted RCTs assessing the effect of LCn3 on PCa outcomes are important as conflicting results from observational studies do not allow conclusions to be drawn so far. In fact, the need for prospective RCTs to identify specific nutrients for PCa patients has recently been highlighted [4]. This trial will contribute to the improvement of available evidence for clinicians and PCa patients who want reliable information on lifestyle strategies to improve survivorship and quality of life [75]. PCa patients would benefit from interventions harbouring few or no side effects. As such, lifestyle and nutritional interventions are appealing as they are innocuous and have the potential to improve various clinically relevant outcomes. High quality trials are important to allow these interventions to be offered to patients in combination with other established treatments.

## Status of the trial

The first patient was enrolled on February 12, 2015. The enrollment and randomization of the planned 130 patients was completed on June 9, 2017. The data collection is expected to end in August 2018.

## Additional file

Additional file 1: Table S1. Trial biospecimen collection. The table contains detailed information on samples with volumes collected and aliquot (DOCX 15 kb)

## Abbreviations

EPIC-26: Expanded Prostate Index Composite score
FACT-COG: Functional Assessment of Cancer Therapy – Cognitive Function
FCRI: Fear of Cancer Recurrence Inventory
FFQ: Food frequency questionnaire
FSI: Fatigue symptoms inventory
HADS: Hospital Anxiety and Depression Scale
HOSO: high oleic acid sunflower oil
IPSS: International Prostate Symptoms Score
LCn3: long-chain omega-3 polyunsaturated fatty acids
LOS: Limit of sensitivity
MAG-EPA: eicosapentaenoic acid monoacylglyceride
OCT: Optimal cutting temperature
PCa: Prostate cancer
RCT: Randomized controlled trial
RP: Radical prostatectomy
SOP: Standard operating procedures
