# Supplementary material for: Effects of concentrated long-chain omega-3 polyunsaturated fatty acid supplementation before radical prostatectomy on prostate cancer proliferation, inflammation, and quality of life: study protocol for a phase IIb, randomized, double-blind, placebo-controlled trial
Source: BMC Cancer. 2018 Jan 10;18:64. doi: 10.1186/s12885-017-3979-9 (PMC5763552; doi:10.1186/s12885-017-3979-9)
Supplement: Additional file 1: Table S1. — Trial biospecimen collection. The table contains detailed information on samples with volumes collected and aliquot (DOCX 15 kb) [file 12885_2017_3979_MOESM1_ESM.docx]

| Sample Type | Volume collected | Aliquot | |
| --- | --- | --- | --- |
|  |  |  |  |
| Blood | 35mL | Plasma | 5 X 1.8mL |
|  |  | Buffy coat | 2 X 0.5mL |
|  |  | Red blood cells | 4 X 0.5mL |
|  |  | Serum | 5 X 1.8mL |
|  |  |  |  |
| Urine post-DRE | 30mL | Crude urine / 50% UTM buffer | 2 X 1.8mL |
|  |  | Crude urine / 50% RNA-Later | 1 X 4.0mL |
|  |  | Crude urine | 1 X 4.0mL + 1 X 2.2 mL |
|  |  | Crude urine / 0.1% ascorbic acid | 1 X 2.2mL |
|  |  | Clarified urine | 1 x 5.4mL |
|  |  | Urine pellet in RNA-Later | pellet + 0.2mL |
|  |  |  |  |
| Tissue | A complete prostate cross-section | Tissue in OCT compound | 1 X each quadrant of the cross-section |
|  |  | Snap Frozen Tissue | 4 X 0.01g |

**Supplementary table 1** Trial biospecimen collection

UTM : Aptima urine Specimen Collection Kit for Male and Female urine Specimens (ref : 301040)

Ascorbic acid: Sigma L-Ascorbic acid (ref: A5960-25g)

RNA later: QIAGEN RNA Stabilization Reagent (ref: 1018087)

OCT: Tissue Tek Oct Compound (ref 62550-12)
